# Innovative and Eco-Friendly Natural Fiber Composites for Dental Impression Materials: A Study on Wheat Bran Reinforcement

**DOI:** 10.3390/polym17040476

**Published:** 2025-02-11

**Authors:** Mohamed A. Aboamer, Abdulmajeed Rasheed Almutairi, Ahmad Alassaf, Tarek M. Alqahtani, Turki F. Almutairi, Ghazwan Najdat Saijari, Nader A. Rahman Mohamed

**Affiliations:** 1 Department of Medical Equipment Technology, College of Applied Medical Sciences, Majmaah University, Majmaah 11952, Saudi Arabia; m.aboamer@mu.edu.sa (M.A.A.);; 2 Department of Biomedical Engineering, Medical City Support Services Management, King Khaled University, Abha 61421, Saudi Arabia; 3 Department of Oral, Maxillofacial and Diagnostic Sciences, College of Dentistry, Majmaah University, Majmaah 11952, Saudi Arabia; 4 Department of Basic Medical Sciences, College of Medicine, Majmaah University, Majmaah 11952, Saudi Arabia; 5 Biomedical Technology Department, College of Applied Medical Sciences in Al-Kharj, Prince Sattam bin Abdulaziz University, Al-Kharj 11942, Saudi Arabia; 6 Biomedical Engineering Department, Faculty of Engineering, Misr University for Science and Technology (MUST), Giza 12568, Egypt

**Keywords:** dental impression materials, wheat bran powder, natural fiber composite, tensile and compression strengths, cost-effectiveness

## Abstract

This study addresses the high cost of traditional dental impression materials by introducing a novel composite material reinforced with wheat bran powder, aiming to reduce expenses while maintaining suitable mechanical performance. Tensile and compression test specimens were prepared according to the ASTM D412 and ASTM D575 standards, respectively, to evaluate the mechanical properties of the pure elastomer and the wheat-bran-reinforced composite. Comparative *t*-tests were conducted to analyze the tensile and compression strengths of both materials, focusing on their cost-effectiveness and suitability for dental applications. The results demonstrate that the wheat-bran-reinforced composite exhibits compression strength (105 MPa) comparable to that of the pure elastomer while offering controlled deformation and enhanced stiffness under compression. Although the composite shows reduced tensile strength (7 MPa vs. 11 MPa), its performance remains adequate for applications requiring moderate tensile properties. Notably, the new material reduces costs by approximately 50%, making it an economical and sustainable alternative for dental impression materials. This innovation aligns with sustainable practices by incorporating natural fibers and offers dentists a cost-effective solution without compromising on performance.

## 1. Introduction

### 1.1. Background

#### 1.1.1. Dental Impression

Dental impression materials play a vital role in creating precise replicas of oral tissues, which are essential for restorative and prosthodontic procedures. These materials are generally classified into two categories: elastic and rigid. Elastic materials, such as hydrocolloids (e.g., agar and alginate) and elastomers (e.g., silicone, polyether, and vinyl polysiloxane), offer excellent detail reproduction and dimensional stability, making them ideal for capturing intricate soft-tissue structures. On the other hand, rigid materials, including dental stone and plaster, are primarily used to accurately replicate hard tissues, such as teeth and bone [1,2,3,4]. Recent advancements in elastic impression materials, such as nanoparticle-infused hydrophilic elastomers and innovative disinfection techniques, have further enhanced their accuracy, stability, and usability [1].

#### 1.1.2. Maximum Human Bite

The maximum human bite force varies across age groups due to factors such as growth and muscle strength. For example, prepubescent males exhibit a bite force of approximately 354.01 ± 134.04 N, whereas adult males demonstrate a force of 284.9 ± 229.57 N. Similarly, females show variability, with prepubescent females generating a bite force of 243.71 ± 84.02 N, which increases to 304.96 ± 115.41 N in adulthood [5,6,7,8,9,10]. These differences are influenced by anatomical, health, and gender-specific factors, underscoring the importance of developing dental materials that can withstand a wide range of forces.

#### 1.1.3. Literature Review

Natural fibers derived from jute, flax, sisal, bamboo, and rice straw are renewable and cost-effective materials with significant biomedical applications due to their antioxidant and antibacterial properties [11,12]. Their performance is influenced by factors such as quality, structure, and environmental conditions like humidity and temperature [13,14,15,16,17]. Bamboo and flax, in particular, are commonly used in surgical meshes, wound dressings, and polymer composites because of their high tensile strength and biocompatibility [18,19,20,21,22,23,24]. Composite materials that combine natural fibers with polymers provide eco-friendly alternatives to synthetic materials, with applications in tissue engineering, drug delivery, and dental technology [25]. However, challenges such as limited biodegradability and insufficient wear resistance hinder their broader adoption [25].

In dental applications, the use of natural fibers in impression materials is rare but holds great promise. These fibers enhance sustainability, reduce costs, and improve mechanical stability. Recent advancements in industries like automotive and aerospace, where natural fibers are increasingly used to reduce weight and energy consumption, highlight their potential for diverse applications, including dentistry [26,27,28,29]. This study aims to explore the integration of natural fibers as reinforcements in dental impression materials to address cost and environmental concerns while maintaining performance standards.

A study titled “Mechanical Properties of Elastomeric Impression Materials: An In Vitro Comparison” evaluated the mechanical characteristics of elastomeric dental impression materials, offering insights into their clinical performance and suitability [30]. The research compared materials such as silicones, polyethers, and polysulfides, examining properties like the elastic modulus, tensile strength, tear strength, and elongation at break through standardized testing methods [31,32,33,34]. The results indicated that heavy-body impression materials exhibit superior tensile strength compared to light-body counterparts [32]. Among light-body materials, hybrid vinylpolyether silicone demonstrated the highest yield strength (2.70 MPa), while polyethers consistently had the lowest tensile strength (1.44 MPa) and yield strength (0.94 MPa). These findings emphasize the importance of selecting elastomeric materials based on their specific physical properties to meet clinical demands.

Light-body vinylpolyether silicone stands out for its exceptional tensile strength, yield strength, and strain characteristics, making it less prone to tearing in narrow interproximal and crevicular regions. The study highlights that materials with higher tear strength and elastic modulus are optimal for capturing detailed impressions in complex anatomical areas, while materials with greater flexibility and elongation at break are more suitable for challenging clinical scenarios.

Another study, “Penetration and Tensile Strength of Various Impression Materials of Vinylsiloxanether, Polyether, and Polyvinylsiloxane”, compared the penetration ability and tensile strength of vinylsiloxanether (VSE), polyether (PE), and polyvinylsiloxane (PVS) dental impression materials [35]. Penetration ability was evaluated using models that simulated gingival sulcus widths (0.05, 0.1, and 0.2 mm) in a moist environment, with each width tested 10 times per material. Extension measurements were conducted with a measuring microscope. Tensile strength was assessed following the ISO 37:2017 and ASTM D412 standards. Statistical analyses included two-way ANOVA and Tukey’s HSD test for penetration ability, as well as one-way ANOVA with Dunnett’s T3 test for tensile strength, with a significance level set at 0.05 [35].

The results showed that polyether exhibited the greatest extension across all simulated sulcus widths, followed by vinylsiloxanether and polyvinylsiloxane. PVS demonstrated significantly higher tensile strength compared to both VSE and PE, while VSE displayed greater tensile strength than PE. Penetration ability was influenced by gingival sulcus width, with wider sulci enabling better penetration. Among the materials, PE had the highest penetration ability, whereas PVS exhibited the greatest tensile strength.

Despite extensive research on elastomeric impression materials, the incorporation of wheat bran powder into dental impression materials and its effects on mechanical properties remain largely unexplored.

### 1.2. Problem Statement

The high cost of traditional dental impression materials in the market poses a challenge for widespread accessibility and cost-effectiveness. To address this, an innovative solution involves reinforcing conventional dental impression materials with wheat bran—a readily available, organic, and natural byproduct commonly used in bread production. This approach aims to significantly reduce the overall cost while maintaining or enhancing the mechanical properties and performance of the dental impression material.

### 1.3. Objectives of the Study

- Develop a novel natural fiber composite material to reduce the cost of traditional dental impression materials.
- Perform a comparative analysis between the pure dental impression material and the newly developed natural fiber composite material, focusing on cost-effectiveness and mechanical properties such as compression and tensile strength.

## 2. Materials and Methods

**Figure 1:**
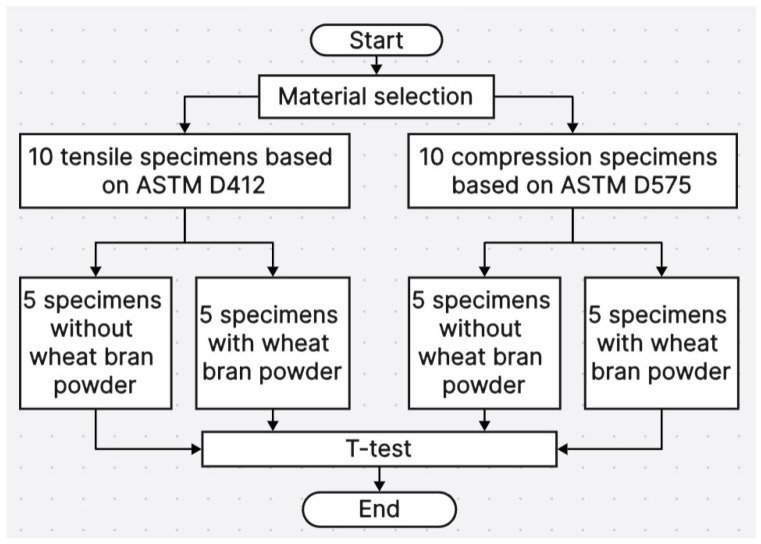
The proposed approach for the whole system.

The proposed approach is divided into four main sections, as illustrated in Figure 1:

1. Material Selection:

The elastomer dental impression material chosen for this study is 3M Espe Express XT Light Body Refill.

2. Fabrication Procedures:

A total of 20 test specimens were fabricated: 10 tensile test specimens, following ASTM D412 standards, and 10 compression test specimens, following ASTM D575 standards.

3. Specimen Partitioning:
  The 10 tensile specimens were divided into two subgroups:
  - Group 1: Five specimens without wheat bran powder.
  - Group 2: Five specimens with wheat bran powder.
  Similarly, the 10 compression specimens were divided into two subgroups:
  - Group 1: Five specimens without wheat bran powder.
  - Group 2: Five specimens with wheat bran powder.

4. Statistical Analysis:

A *t*-test was employed to determine if there were any significant differences in tensile and compression properties between the subgroups before and after incorporating wheat bran powder.

### 2.1. Fabrication Procedures

**Figure 2:**
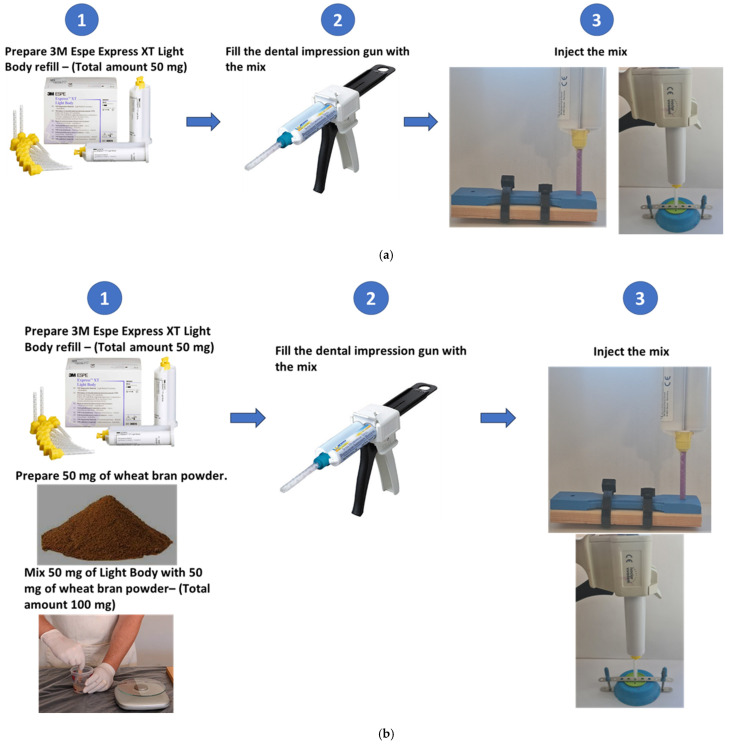
Steps of preparing the pure material and the new mixture (**a**) for pure material and (**b**) for the mixture.

The pure material selected for this study was 3M Espe Express XT Light Body Refill [36]. Five tensile specimens were fabricated using the pure material, as shown in Figure 2a. Additionally, five tensile specimens were fabricated by mixing 50 mg of wheat bran powder with 50 mg of Light Body, as shown in Figure 2b.

#### 2.1.1. Fabrication of ASTM D412 Tensile Specimen Mold

**Figure 3:**
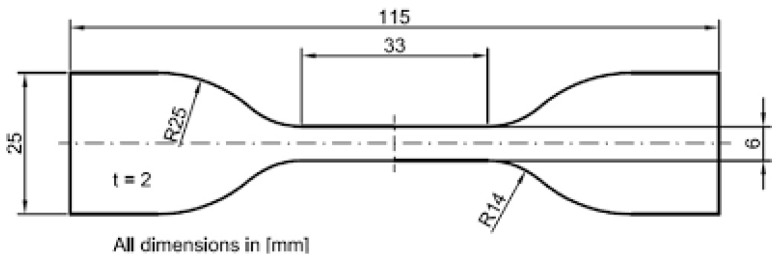
Tensile test specimen ASTM D412.

The shapes and key geometrical dimensions of the ASTM D412 [37,38] tensile test specimens, as illustrated in Figure 3, were utilized to design the molds (negative parts) required for ASTM D412 testing. These molds were fabricated using a 3D printer.

The fabricated molds were used to produce specimens for evaluating the maximum stress and load capacities of the silicone rubber composite, as well as for assessing its overall mechanical properties.

**Figure 4:**
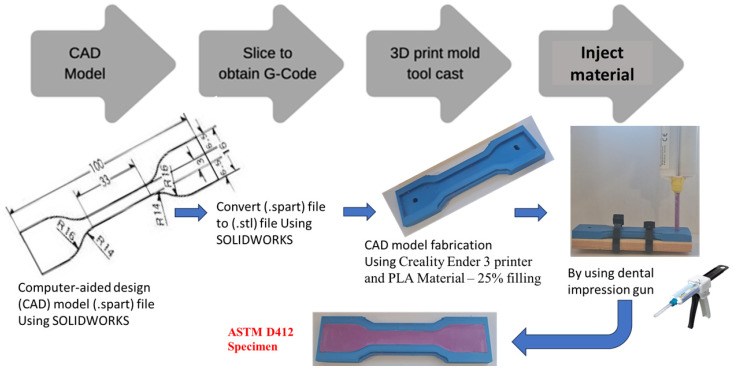
Detailed steps about how to fabricate 10 tensile specimens.

The detailed process of fabricating five tensile specimens from the pure material and an additional five tensile specimens from the new mixture is outlined in Figure 4. First, the mechanical drawing was created using SOLIDWORKS software (Premium 2020 SP0.0) and saved in spart-file format. Next, the part file was converted into G-code or STL-file format to ensure compatibility with the 3D printer. In the third step, a prototype model was produced using a Creality Ender 3 printer, utilizing PLA material with 25% infill. Finally, the material was injected into the print mold tool, resulting in specimens that conform to ASTM D412 standards.

**Figure 5:**
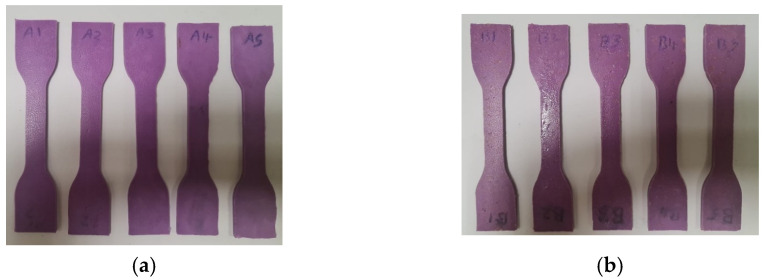
Tensile test specimens (ASTM D412): (**a**) 5 specimens without wheat bran powder, (**b**) 5 specimens with wheat bran powder.

The fabricated tensile specimens are illustrated in Figure 5, where (Figure 5a) shows the five specimens without wheat bran powder, and (Figure 5b) shows the five specimens with wheat bran powder.

#### 2.1.2. Fabrication of ASTM D575 Compression Test Specimen Mold

The ASTM D575 [39,40,41] standard specifies the required dimensions for test specimens used in dental impression material testing. Each specimen must be a cylindrical shape with a diameter of 28.4 mm and a height of 12.5 mm. The top and bottom surfaces of the cylinder must be flat, and the sides must remain parallel. These specifications were used to design the molds (negative parts) needed for ASTM D575 testing, which were fabricated using a 3D printer.

**Figure 6:**
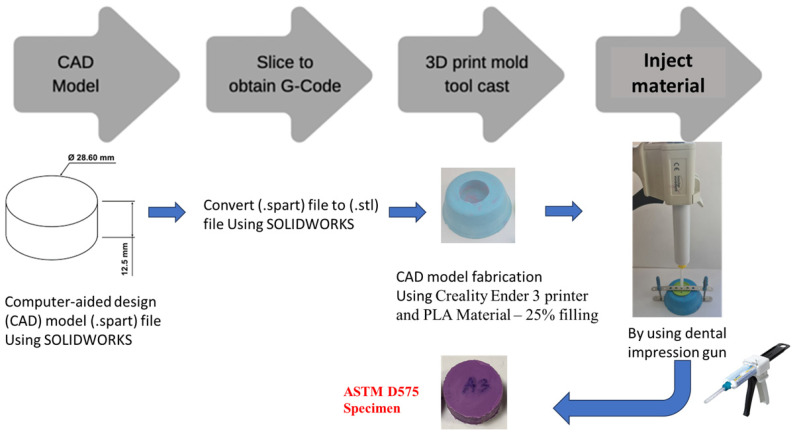
Detailed steps about how to fabricate 10 compression specimens.

The process of creating five compression specimens from the pure material, along with an additional five specimens from the new mixture, is shown in Figure 6. First, a mechanical drawing was prepared in SOLIDWORKS software and saved as a part file. This file was then converted into G-code or STL format to ensure compatibility with the 3D printer. Next, a prototype model was fabricated using a Creality Ender 3 printer with PLA material at a 25% infill ratio. Finally, the material was injected into the 3D-printed mold tool, producing specimens that fully comply with ASTM D575 standards.

**Figure 7:**
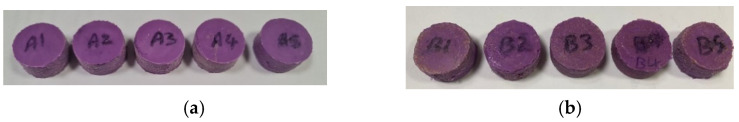
Compression test specimens (ASTM D695): (**a**) 5 specimens without wheat bran powder, (**b**) 5 specimens with wheat bran powder.

The fabricated compression specimens are illustrated in Figure 7, where (Figure 7a) shows the five specimens without wheat bran powder, and (Figure 7b) shows the five specimens with wheat bran powder.

### 2.2. Three-Dimensional Printer

A 3D printer is an automated device controlled through a computer that creates three-dimensional objects through a process known as additive manufacturing, in which material is added layer by layer. This technology has revolutionized numerous industries, enabling rapid prototyping, personalized manufacturing, and the creation of intricate shapes that would be difficult or impossible to achieve with conventional manufacturing methods [42,43].

#### Principle of Operation

The fundamental principle of 3D printing involves the gradual addition of material layer by layer to build an object. This contrasts with subtractive manufacturing techniques, such as machining, where material is removed from a solid block to achieve the desired shape [42,44].

### 2.3. t-Test

The *t*-test is a statistical hypothesis testing method used to determine whether there is a significant difference between the means of two groups. It is widely used in various research fields, including medicine, psychology, and social sciences. The *t*-test is a valuable tool that helps researchers draw conclusions about group differences. This literature review will discuss the *t*-test, its different types, and provide references to recent studies that have employed this statistical method. Additionally, the equation for the *t*-test will be included to help readers understand the statistical calculations involved.

The *t*-test compares the means of two groups to assess whether the difference between them is statistically significant. It helps determine if the observed difference in means is due to chance or represents a true difference between the groups. There are several types of *t*-tests, including the independent samples *t*-test, paired samples *t*-test, and one-sample *t*-test. The independent samples *t*-test is used when the two groups being compared are independent of one another, while the paired samples *t*-test is used when the two groups are related or paired.

#### *t*-Test Equation

The equation for the *t*-test depends on the type of *t*-test being used. The general equation for the *t*-test is presented in Equation (1) [45,46]:

(1) $$t=\frac{\left(\bar{x_{1}}-\bar{x_{2}}\right)}{s\sqrt{\left(\frac{1}{n_{1}}+\frac{1}{n_{2}}\right)}}$$

where

*t* is the *t*-test statistic;

$\bar{x_{1}}$ is the mean of the first group;

$\bar{x_{2}}$ is the mean of the second group;

s is the pooled standard deviation of the two groups;

$n_{1}$ is the sample size of the first group;

$n_{2}$ is the sample size of the second group.

## 3. Results

### 3.1. Universal Testing Machine

**Figure 8:**
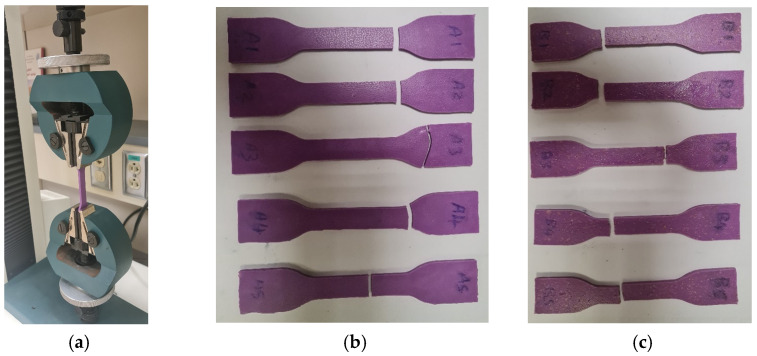
Tensile test. (**a**) Universal testing machine, (**b**) tensile specimens without wheat bran powder, and (**c**) tensile specimens with wheat bran powder.

After performing the tensile test using a universal testing machine [47], key tensile parameters, including tensile strength, ultimate stress, fracture stress, and forces, were measured [48,49]. Two distinct groups of tensile specimens were tested in accordance with ASTM D412 standards, as shown in Figure 8a. Figure 8b,c illustrate the tensile specimens before and after the incorporation of wheat bran powder, respectively. Specifically, Figure 8b depicts the specimens without wheat bran powder, while Figure 8c shows the specimens with wheat bran powder.

### 3.2. Compression Test Specimens

**Figure 9:**
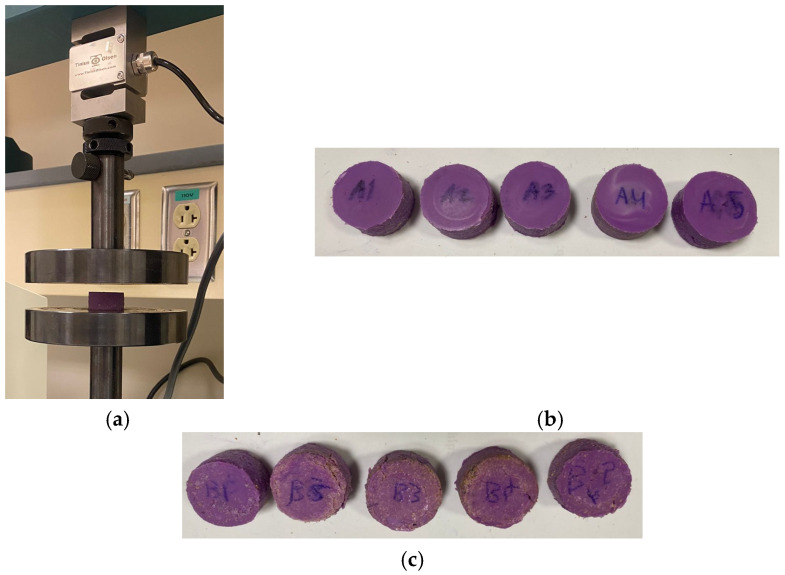
Compression test. (**a**) Universal testing machine, (**b**) tensile specimens without wheat bran powder, and (**c**) tensile specimens with wheat bran powder.

The universal testing machine was used to measure compression parameters, including yield strength, ultimate stress, fracture stress, and forces, for two distinct groups of specimens in accordance with ASTM D575 standards, as shown in Figure 9a. Figure 9b,c illustrate the specimens before and after the incorporation of wheat bran powder, respectively. Specifically, Figure 9b depicts the specimens without wheat bran powder, while Figure 9c presents the specimens with wheat bran powder.

### 3.3. Tensile Test

#### 3.3.1. Pure Elastomer Without Wheat Bran Powder

**Figure 10:**
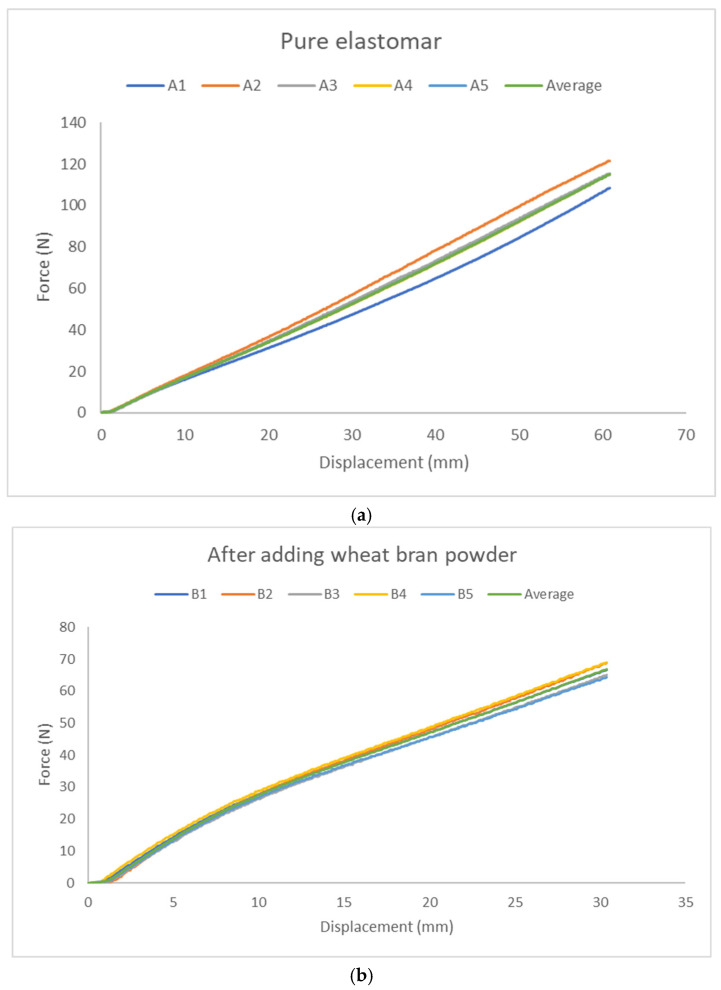
Force–displacement curves: (**a**) five pure Elastomer specimens and their average curve, (**b**) five specimens and their average curve.

This study investigated potential changes in the mechanical properties of specimens with and without the addition of wheat bran powder. Each group consisted of five specimens, with force–displacement data for each specimen and the group averages depicted in Figure 10a. The x-axis represents displacement (mm), and the y-axis represents force (N).

According to ASTM D412 standards, key parameters include yield force (N), fracture force (N), and ultimate force, which coincides with the fracture point.

#### 3.3.2. Elastomer Mixed with Wheat Bran Powder

The goal here was to analyze how the addition of wheat bran powder affects mechanical properties. Five specimens were fabricated for this group, and Figure 10b shows the force–displacement data for individual specimens and the group average. As with the pure elastomer group, displacement is plotted on the x-axis, and force is plotted on the y-axis.

Similarly to the pure material, ASTM D412 standards apply, and the yield force, fracture force, and ultimate force are represented at the fracture point.

#### 3.3.3. Comparison Before and After Adding Wheat Bran Powder

**Figure 11:**
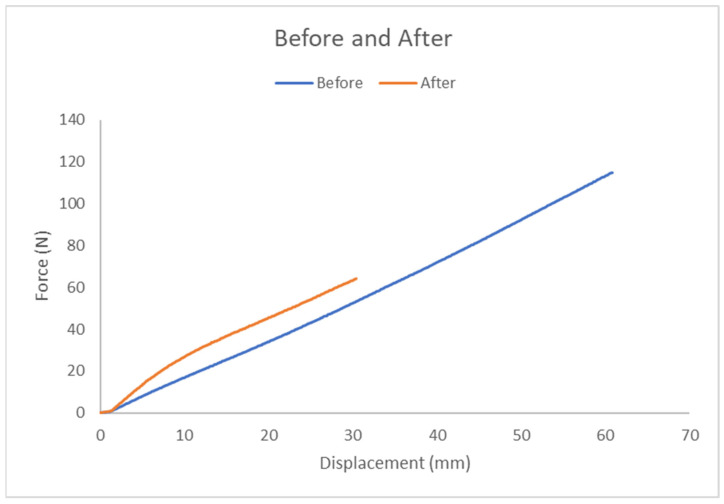
Force–displacement for average curves before and after adding wheat bran powder.

A comparison of tensile test results is illustrated in Figure 11. The pure group exhibited greater displacement capacity, while the wheat bran powder group supported higher forces.

Table 1 illustrates the compression test parameters (fracture force and displacement) between the two groups:

- With Wheat Bran Powder: ~66.8 ± 2.2 N; displacement ~30.4 ± 0 mm.
- Without Wheat Bran Powder: ~115 ± 4.6 N; displacement ~60.8 ± 0 mm.

This indicates that the pure elastomer group sustains higher displacement, whereas the wheat bran powder group supports greater force.

#### 3.3.4. *t*-Test for Tensile Test Specimens

**Figure 12:**
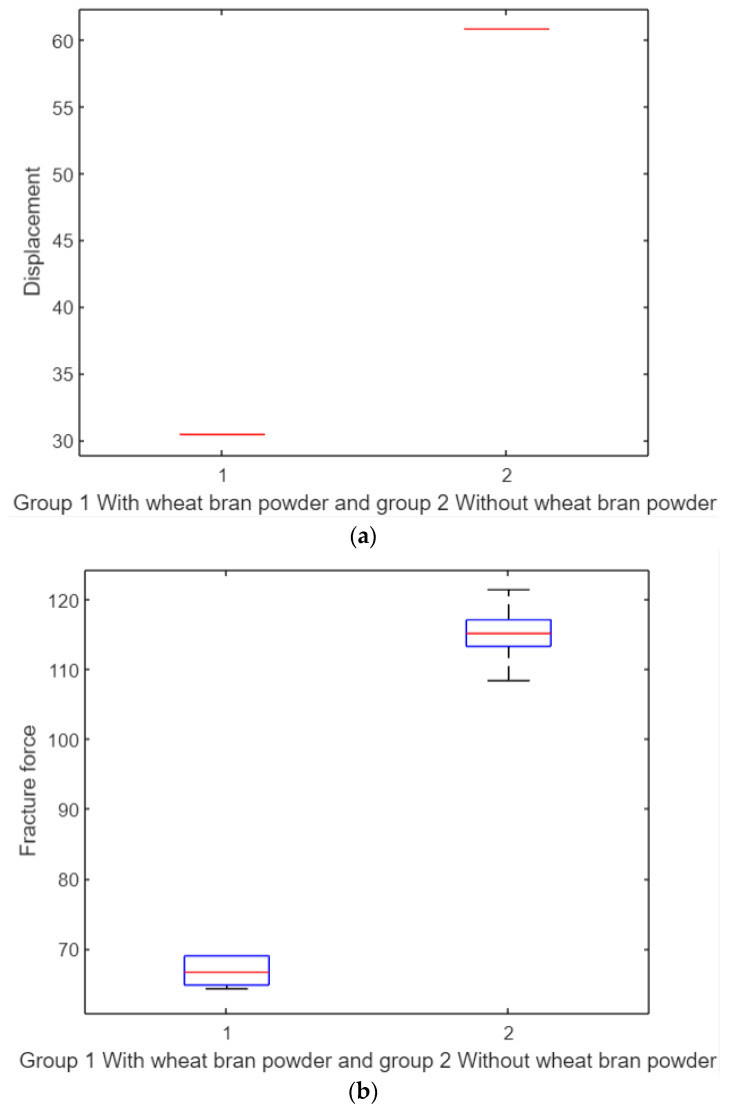
*t*-test. (**a**) Clear significant difference between the displacement parameter of the two groups. (**b**) Clear significant difference between the tensile fracture force parameter of the two groups.

Applying a *t*-test (Figure 12a) revealed a significant difference in displacement between the two groups, with a *p*-value of 0. The addition of wheat bran powder results in a 50% reduction in tensile displacement properties. Similarly, for tensile fracture force (Figure 12b), the *t*-test demonstrated a significant difference, with the wheat bran powder group showing a 50% decrease in tensile properties compared to the pure group.

As shown in Figure 12a, the box plot represents the displacement results for two groups:

- Group 1: With wheat bran powder.
- Group 2: Without wheat bran powder.

The red line in the middle of each box plot represents the median of the displacement data. In this figure, the red line appears as a single horizontal line because the data distribution within each group is extremely narrow, indicating minimal variation in displacement. This small variation causes the box to collapse, making the median line appear as a single flat line.

In contrast, Figure 12b presents the box plot for the fracture force results of the same two groups:

- Group 1: With wheat bran powder.
- Group 2: Without wheat bran powder.

Here, the red line represents the median of the fracture force data. Unlike Figure 12a, the red line does not appear as a single flat line because the data in each group exhibit greater variability. Consequently, the box plot displays a clear distribution of values, including the interquartile range (box), whiskers, and potential outliers.

#### 3.3.5. Average Stress–Strain Curves for Both Groups

**Figure 13:**
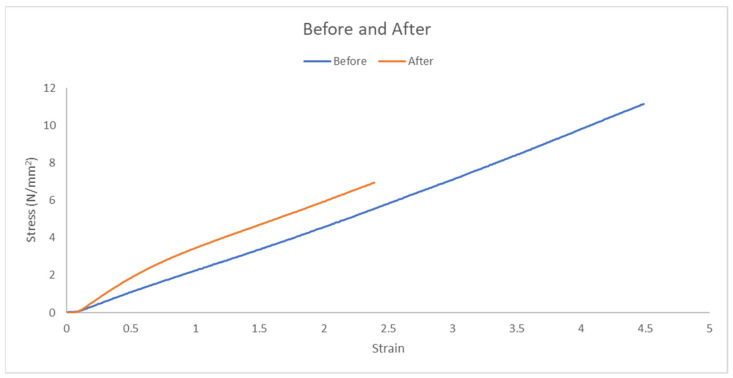
Stress–strain for average curves before and after adding wheat bran powder.

Figure 13 shows the average stress–strain curves for both groups, as shown in Table 2:

- With Wheat Bran Powder: Fracture stress ~7 MPa, fracture strain ~2.5.
- Without Wheat Bran Powder: Fracture stress ~11 MPa, fracture strain ~4.5.

The tensile test results for the two groups—elastomer composite with wheat bran powder and pure elastomer composite—demonstrate notable differences in mechanical performance. The strain values for the group with wheat bran powder averaged 2.5, while the pure elastomer group exhibited a significantly higher strain average of 4.5. Similarly, the stress values for the group with wheat bran powder were 7 MPa compared to 11 MPa for the pure elastomer group.

These results suggest that the incorporation of wheat bran powder reduces the strain and stress capacities of the composite material. The pure elastomer group, with its higher strain and stress values, shows greater tensile capacity and elongation, making it more suited for applications requiring higher flexibility and tensile strength. In contrast, the wheat bran powder composite, though demonstrating lower tensile properties, may be advantageous in applications where controlled deformation and moderate stress-handling capabilities are sufficient.

The reduced strain and stress in the wheat bran powder group may be attributed to the interaction between the wheat bran particles and the elastomer matrix. The presence of wheat bran likely introduces localized stiffness, restricting the material’s ability to elongate under tensile load. Additionally, the lower stress values in this group may indicate a compromise in the material’s load-bearing capacity due to the inclusion of the filler.

Despite these reductions in tensile properties, the wheat bran powder composite offers potential benefits in terms of cost-effectiveness and application-specific performance. The addition of wheat bran powder reduces material costs while maintaining sufficient mechanical properties for applications that do not require extreme tensile strength or elongation. Such applications might include load-bearing components in moderate-stress environments or eco-friendly alternatives for non-critical structural uses.

In conclusion, while the pure elastomer composite demonstrates superior tensile strength and flexibility, the wheat bran powder composite provides a balanced trade-off between performance and cost, making it a viable option for specific applications where moderate mechanical properties and economic feasibility are prioritized.

### 3.4. Compression Test

#### 3.4.1. Pure Elastomer Without Wheat Bran Powder

**Figure 14:**
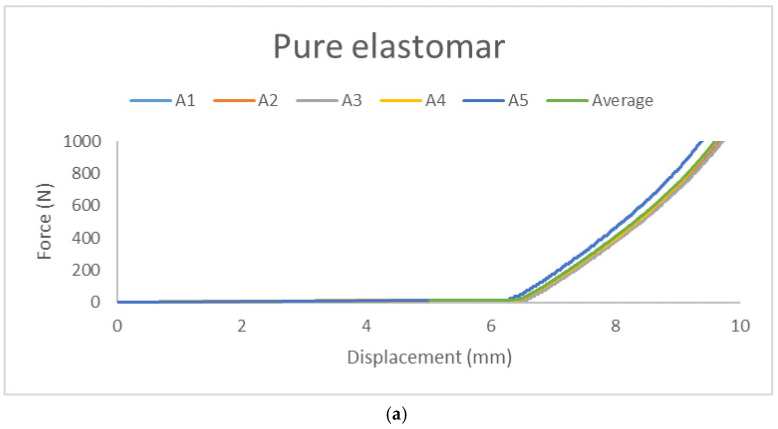

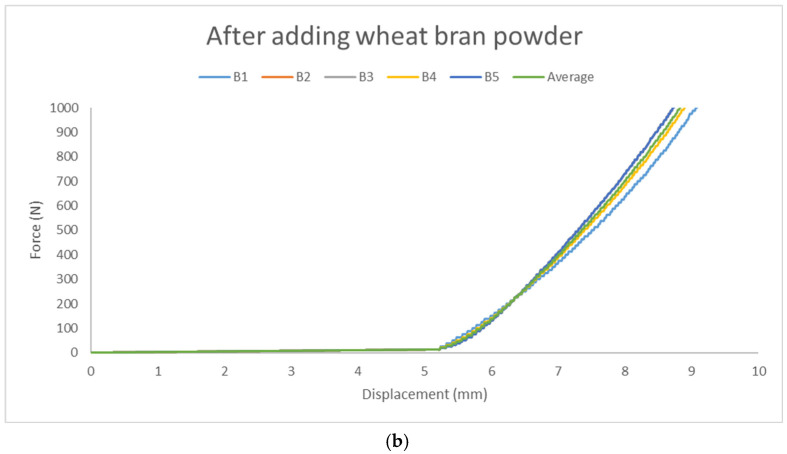
Force–displacement curves: (**a**) five specimens and average curve, (**b**) five specimens and average curve.

This section examines changes in the compression properties of specimens. Force–displacement data for the five specimens and their average are shown in Figure 14a. The x-axis represents displacement (mm), and the y-axis represents force (N).

According to ASTM D575 standards, parameters include yield force (N), fracture force (N), and ultimate force, which converge at the fracture point.

#### 3.4.2. Elastomer Mixed with Wheat Bran Powder

This section examines changes in the compression properties of specimens. Force–displacement data for the five specimens and their average are shown in Figure 14b. The x-axis represents displacement (mm), and the y-axis represents force (N).

According to ASTM D412 standards, parameters include yield force (N), fracture force (N), and ultimate force, which coincide with the fracture point.

#### 3.4.3. Comparison Before and After Adding Wheat Bran Powder

**Figure 15:**
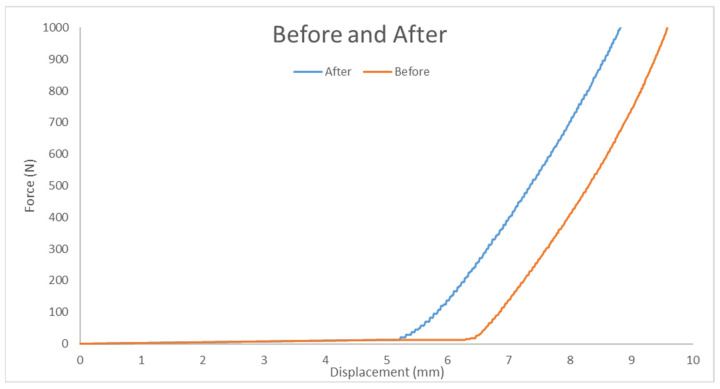
Force–displacement for average curves before and after adding wheat bran powder.

Figure 15 illustrates the comparison of results as follows:

- Pure Elastomer Group: Supports greater displacement.
- Wheat Bran Powder Group: Sustains higher force in the displacement range of 5.5–9.5 mm.

Fracture Force and Displacement as shown in Table 3:

- With Wheat Bran Powder: Fracture force ~1000 ± 0 N; displacement ~9.06 ± 0 mm.
- Without Wheat Bran Powder: Fracture force ~1000 ± 0 N; displacement ~9.6 ± 0.13 mm.

As shown in Table 3, the fracture force for the group with wheat bran powder is roughly 1000 ± 0 N, with a fracture displacement of around 9.06 ± 0 mm. Conversely, the fracture force for the group without wheat bran powder is approximately 1000 ± 0 N, with a fracture displacement of about 9.6 ± 0.13 mm.

This suggests that the pure group shows slightly higher displacement, but both groups sustain similar fracture forces within the range of human bite forces.

#### 3.4.4. *t*-Test for Compression Test Specimens

**Figure 16:**
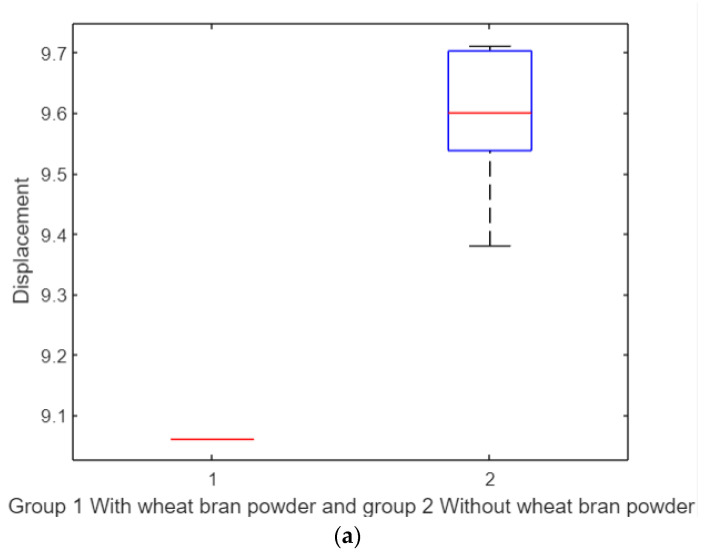

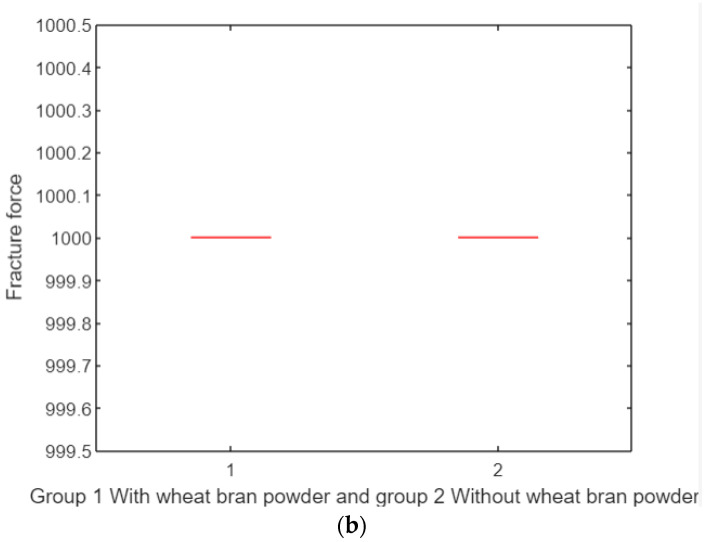
*t*-test. (**a**) A significant difference between the displacement parameter of the two groups. (**b**) No significant difference between the tensile fracture force parameter of the two groups.

The *t*-test (Figure 16a) demonstrated a statistically significant difference in displacement between the two groups (*p*-value = 0.000018). However, the reduction in displacement for the wheat bran powder group is minimal (~0.6 mm). The *t*-test for fracture force (Figure 16b) found no significant differences (*p*-value = 0). The new material performs comparably to the pure elastomer in terms of load-bearing capacity, making it suitable for dental applications.

#### 3.4.5. Average Stress–Strain Curves for Both Groups

**Figure 17:**
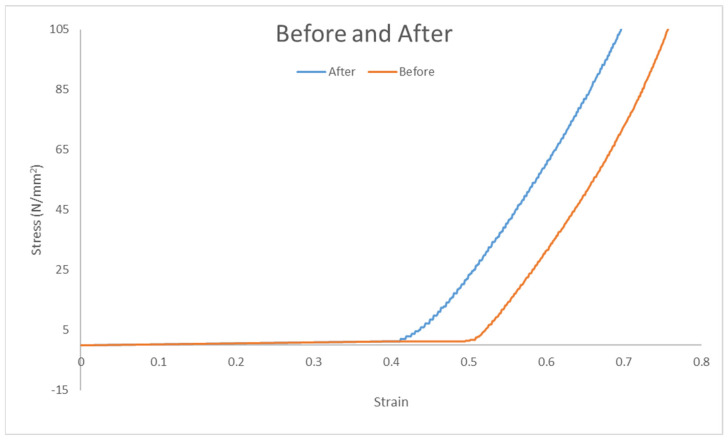
Stress–strain for average curves before and after adding wheat bran powder.

Figure 17 shows the average stress–strain curves, and Table 4 illustrates the compression test parameters between two groups:

- With Wheat Bran Powder: Fracture stress ~105 MPa, fracture strain ~0.7.
- Without Wheat Bran Powder: Fracture stress ~105 MPa, fracture strain ~0.78.

The results of the mechanical testing for the two groups—elastomer composite with wheat bran powder and the pure elastomer composite—show interesting trends. The strain values for the group containing wheat bran powder averaged 0.7, compared to 0.78 for the group without wheat bran powder. However, both groups demonstrated identical average stress values of 105 MPa. This suggests that while the pure elastomer exhibits a higher strain capacity, the wheat bran powder group maintains comparable stress-bearing capabilities.

The strain–stress relationship highlights the ability of the wheat bran powder group to endure higher stress levels in the strain range of 0.4–0.7. This indicates that the addition of wheat bran powder enhances the material’s ability to withstand stress under lower strain conditions, which is critical for applications requiring consistent stress resistance without excessive elongation.

#### 3.4.6. Maximum Human Bite Before and After Adding Wheat Bran Powder

As discussed, the maximum human bite force varies across age groups due to factors such as growth, muscle development, and changes in muscle strength [2]. In this context, the new elastomer composite with wheat bran powder offers significant advantages. Despite its slightly reduced strain capacity compared to the pure elastomer, the composite material demonstrates comparable force and stress support. Moreover, the incorporation of wheat bran powder reduces production costs by approximately 50%, making it a cost-effective alternative for dental applications.

This cost reduction, coupled with comparable performance, positions the wheat bran powder composite as a viable material for bite-force support devices. Its stress-handling capabilities make it particularly suitable for applications involving adult males, females, and young adults, where high stress resistance is required without the need for excessive tensile displacement.

**Figure 18:**
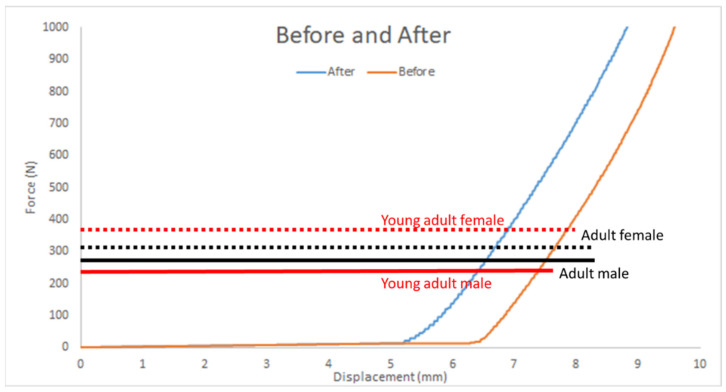
The average bite for adult and young adults for female and males on force–displacement for average curves before and after adding wheat bran powder.

In summary, the addition of wheat bran powder to the elastomer composite optimizes the material’s performance in specific stress–strain conditions while providing economic benefits, making it a promising option for use in dental and other mechanical applications as shown in Figure 18.

Both materials have maximum force capacities that exceed typical human bite forces, which vary across age groups and genders. This ensures that the materials can handle the stress exerted during dental procedures without failure.

The materials’ properties, especially their ability to support forces up to 1000 N, make them reliable options for dental applications.

### 3.5. Comparison Between Tensile Test and Compression Test

The tensile and compression test results for the elastomer composites, both with and without wheat bran powder, highlight distinct mechanical behaviors under different loading conditions.

#### 3.5.1. Tensile Test Results

##### With Wheat Bran Powder

- Average Strain: 2.5
- Average Stress: 7 MPa

##### Without Wheat Bran Powder

- Average Strain: 4.5
- Average Stress: 11 MPa

The tensile test demonstrates that the pure elastomer composite has superior elongation (strain) and tensile strength (stress) compared to the wheat bran powder composite. The inclusion of wheat bran powder introduces stiffness, reducing the material’s ability to stretch under tensile loads. This makes the pure elastomer more suitable for applications requiring high flexibility and tensile capacity, whereas the wheat bran powder composite is better suited for applications with moderate tensile demands and cost-effectiveness.

#### 3.5.2. Compression Test Results

##### With Wheat Bran Powder

- Average Strain: 0.7
- Average Stress: 105 MPa

##### Without Wheat Bran Powder

- Average Strain: 0.78
- Average Stress: 105 MPa

The compression test shows similar stress values for both groups, but the strain capacity of the wheat bran powder composite is slightly lower than that of the pure elastomer. The results suggest that the wheat bran powder composite performs comparably in terms of stress resistance under compression, despite reduced strain. This indicates that the wheat bran filler enhances the material’s load-bearing capability under compression by providing structural support and minimizing excessive deformation.

#### 3.5.3. Key Comparisons

##### Stress Performance

- In tensile tests, the pure elastomer (11 MPa) significantly outperforms the wheat bran powder composite (7 MPa).
- In compression tests, both materials achieve identical stress values (105 MPa), demonstrating that the addition of wheat bran powder does not compromise the stress-handling capacity under compression.

##### Strain Capacity

- In both tests, the pure elastomer exhibits higher strain values, indicating greater flexibility and elongation under load.
- The wheat bran powder composite shows reduced strain in both tensile (2.5 vs. 4.5) and compression (0.7 vs. 0.78) tests, highlighting its stiffness due to the filler.

#### 3.5.4. Material Suitability

- Pure Elastomer: Performs better in tensile applications requiring high flexibility and strength, such as stretchable components and high-stress tensile environments.
- Wheat Bran Powder Composite: Performs well in compression-based applications where deformation control and cost-effectiveness are key priorities, such as load-bearing supports or compression-resistant structures.
- The comparison between the tensile and compression tests highlights the trade-offs introduced by incorporating wheat bran powder into the elastomer. While the wheat bran powder composite exhibits reduced tensile properties, it maintains comparable compression stress and provides better stiffness control. This makes the wheat bran powder composite a viable and cost-effective alternative for applications involving compression, whereas the pure elastomer remains preferable for tensile applications requiring higher flexibility and strength.

## 4. Discussion

The findings of this study offer valuable insights into the mechanical performance of wheat bran reinforced dental impression materials compared to pure elastomer composites. By tackling the issue of high-cost traditional dental impression materials, this study highlights the potential of wheat bran as a cost-effective and sustainable reinforcement material.

### 4.1. Mechanical Behavior and Performance

The tensile test results reveal that the pure elastomer composite demonstrates superior elongation (strain: 4.5) and tensile strength (stress: 11 MPa) compared to the wheat-bran-reinforced composite (strain: 2.5, stress: 7 MPa). These differences highlight the trade-offs associated with the addition of wheat bran powder. While the filler material enhances stiffness, it limits the elastomer’s ability to stretch under tensile loads. Consequently, the pure elastomer is better suited for applications requiring high flexibility and tensile capacity, such as stretchable components. In contrast, the wheat-bran-reinforced composite provides adequate tensile properties for applications with moderate performance demands while offering the added benefit of significant cost savings.

In compression tests, both materials exhibit comparable stress-handling capacities (105 MPa), ensuring their suitability for dental impression applications where compression forces typically remain below 1000 N. However, the wheat-bran-reinforced composite demonstrates a slightly reduced strain capacity (0.7 vs. 0.78), reflecting its enhanced stiffness and controlled deformation under compression. This increased stiffness makes the wheat-bran-reinforced composite particularly suitable for load-bearing applications and situations requiring controlled, moderate deformation.

### 4.2. Why Wheat Bran Improves Properties

Wheat bran enhances the mechanical properties of the composite by serving as a structural reinforcement within the elastomer matrix. Its fibrous nature contributes to localized stiffness, limiting elongation under tensile loads while improving the material’s resistance to compression forces. Moreover, the irregularities and microstructure of the wheat bran promote better load distribution within the composite, particularly under compression, reducing excessive deformation and increasing the material’s durability in such applications.

### 4.3. Composition of Wheat Bran

The mechanical advantages of wheat bran can be attributed to its composition, which includes cellulose, hemicellulose, lignin, and protein—key structural compounds:

- Cellulose provides high tensile strength and stiffness, enhancing the composite’s structural integrity.
- Hemicellulose facilitates bonding with the elastomer matrix due to its partially amorphous nature.
- Lignin contributes to increased stiffness and resistance to mechanical stress.
- Proteins and other minor components improve adhesion and interaction within the elastomer matrix, further reinforcing the material.

Together, these compounds create a synergistic effect, enabling the composite to efficiently withstand compressive loads while simultaneously reducing material costs.

### 4.4. Suitability for Dental Applications

The wheat-bran-reinforced composite meets the mechanical requirements for dental impression materials. Its capacity to withstand forces exceeding typical human bite forces across various age groups and genders ensures reliability in dental applications. Additionally, while its reduced tensile properties are adequate for moderate-demand applications, its superior performance under compression makes it especially suitable for load-bearing dental impression tasks.

### 4.5. Broader Implications

This study demonstrates that wheat bran serves as a sustainable and cost-effective reinforcement for elastomer composites. By lowering material costs by approximately 50%, the wheat-bran-reinforced composite addresses the economic challenges associated with traditional dental impression materials. Its alignment with sustainable practices, through the utilization of a natural byproduct, further highlights its potential as an eco-friendly alternative. Beyond dentistry, the findings suggest broader applications in industries requiring moderate tensile properties and high compression resistance, such as packaging, construction, and biomedical devices.

## 5. Conclusions

This study successfully developed a cost-effective and sustainable dental impression material by reinforcing elastomers with wheat bran, a readily available byproduct. The composite achieves a significant cost reduction (~50%) while maintaining mechanical properties suitable for dental applications. The pure elastomer exhibits superior tensile strength and flexibility, making it ideal for applications requiring high elongation. In contrast, the wheat bran composite, with its enhanced stiffness and deformation control, is well suited for compression-based applications. Both materials meet the mechanical requirements for dental impression use, demonstrating their potential as viable alternatives to traditional materials.

## 6. Future Work

Future studies can focus on optimizing the composition of wheat bran powder to achieve a better balance between strength and flexibility. A detailed analysis of the material’s structure could involve techniques such as Energy-Dispersive X-ray Spectroscopy (EDS/EDX) to examine its elemental composition and surface topography to assess its structural characteristics. Additionally, research could explore the material’s long-term performance under repeated use, various environmental conditions, and aging to evaluate its effectiveness and durability in real-world applications.

## Figures and Tables

**Table 1:** Displacement-Force tensile test parameters between the two groups.

Groups		Displacement (mm)	Force (N)
With wheat bran powder	B1	30.4	66.65
	B2	30.4	69
	B3	30.4	65
	B4	30.4	69
	B5	30.4	64.3
	Average	30.4 ± 0	66.8 ± 2.2
Without wheat bran powder	A1	60.825	108.34
	A2	60.825	121.35
	A3	60.825	115.63
	A4	60.825	114.9
	A5	60.825	115.1
	Average	60.8 ± 0	115 ± 4.6

**Table 2:** Average Stress-Stain tensile test parameters between the two groups.

Groups	Average	
	Strain	Stress (Mpa)
With wheat bran powder	2.5	7
Without wheat bran powder	4.5	11

**Table 3:** Displacement-Force compression test parameters between the two groups.

Groups		Displacement (mm)	Force (N)
With wheat bran powder	B1	9.06	1000
	B2	9.06	1000
	B3	9.06	1000
	B4	9.06	1000
	B5	9.06	1000
	Average	9.06 ± 0	1000 ± 0
Without wheat bran powder	A1	9.6	1000
	A2	9.7	1000
	A3	9.71	1000
	A4	9.59	1000
	A5	9.38	1000
	Average	9.6 ± 0.13	1000 ± 0

**Table 4:** Average Stress-Stain compression test parameters between the two groups.

Groups	Average	
	Strain	Stress (Mpa)
With wheat bran powder	0.7	105
Without wheat bran powder	0.78	105

## Data Availability

The original contributions presented in this study are included in the article. Further inquiries can be directed to the corresponding author.
